# Real-World Research on Retinal Diseases Using Health Claims Database: A Narrative Review

**DOI:** 10.3390/diagnostics14141568

**Published:** 2024-07-19

**Authors:** Seong Joon Ahn

**Affiliations:** Department of Ophthalmology, Hanyang University Hospital, Hanyang University College of Medicine, Seoul 04763, Republic of Korea; ahnsj81@gmail.com; Tel.: +82-2-2290-8574

**Keywords:** retinal diseases, real-world research, health claims database, future directions

## Abstract

Real-world data (RWD) has emerged as a crucial component in understanding and improving patient outcomes across various medical conditions, including retinal diseases. Health claims databases, generated from healthcare reimbursement claims, offer a comprehensive source of RWD, providing insights into patient outcomes, healthcare utilization, and treatment effectiveness. However, the use of these databases for research also presents unique challenges. This narrative review explores the role of real-world research on retinal diseases using health claims databases, highlighting their advantages, limitations, and potential contributions to advancing our understanding and management of the diseases. The review examines the applications of health claims databases in retinal disease research, including epidemiological studies, comparative effectiveness and safety analyses, economic burden assessments, and evaluations of patient outcomes and quality of care. Previous findings demonstrate the value of these databases in generating prevalence and incidence estimates, identifying risk factors and predictors, evaluating treatment effectiveness and safety, and understanding healthcare utilization patterns and costs associated with retinal diseases. Despite their strengths, health claims databases face challenges related to data limitations, biases, privacy concerns, and methodological issues. Accordingly, the review also explores future directions and opportunities, including advancements in data collection and analysis, integration with electronic health records, collaborative research networks and consortia, and the evolving regulatory landscape. These developments are expected to enhance the utility of health claims databases for retinal disease research, resulting in more comprehensive and impactful findings across diverse retinal disorders and robust real-world insights from a large population.

## 1. Introduction

In the evolving landscape of healthcare research, the utilization of real-world data (RWD) has emerged as a pivotal element in understanding and improving patient outcomes across various medical conditions. Real-world data, encompassing information collected from sources outside of traditional clinical trials, offers a comprehensive view of patient health status, treatment modalities, and healthcare utilization in routine clinical practice [1,2,3]. Among the diverse sources of RWD, health claims databases stand out due to their extensive coverage and depth of information [4]. These databases, generated from healthcare providers’ requests for payment to health plans, provide invaluable insights into patient outcomes, healthcare utilization, and treatment effectiveness [4]. However, while the breadth and accessibility of health claims databases offer significant advantages for research, they also present unique challenges, including limitations in clinical detail and potential biases inherent in claims data [5].

Retinal diseases exemplify conditions where RWD and health claims databases can significantly contribute to our understanding and management strategies [6]. Retinal diseases encompass a wide range of disorders, including diabetic retinopathy (DR), retinopathy of prematurity, and age-related macular degeneration, each with distinct etiologies, pathophysiologies, and treatment approaches. The global burden of retinal diseases, particularly DR, underscores the importance of population-based research in identifying trends, evaluating treatment outcomes, and understanding disease progression on a large scale [7].

The purpose of this review is to explore the role of health claims databases and their findings in real-world research on retinal diseases. By examining the advantages and limitations of employing health claims databases for research, this review aims to highlight the significance of RWD in advancing our knowledge of retinal diseases. Furthermore, the scope of this review encompasses the challenges faced in utilizing health claims databases, and the potential for these databases to inform clinical practice and policy making in the management of retinal diseases. Through this comprehensive examination, we seek to elucidate the contributions of health claims databases to retinal disease research and the broader implications for patient care and healthcare systems.

## 2. Overview of Health Claims Databases

Health claims databases are extensive electronic repositories of patient data collected primarily for administrative and billing purposes by healthcare payers, including government programs and private insurance companies [4,5,8]. These databases provide a wealth of information on healthcare utilization, costs, and outcomes, making them invaluable for real-world research [4,5].

The data in clinical registries, such as the Intelligent Research in Sight (IRIS) registry, may include detailed information on disease diagnoses, treatments, patient outcomes, and follow-up care, mostly derived from electronic health records (EHRs) of participating ophthalmology practices. These registries help identify the best practices and improve patient care in ophthalmology. In contrast, health claims databases are primarily used to track financial transactions and reimbursements related to healthcare services. They collect information about patient demographics, diagnoses, procedures, and payment details submitted by healthcare providers to insurers for reimbursement. These databases are particularly useful for health economics research and policy analysis. However, they are widely used in ophthalmology literature to obtain real-world data on healthcare use and practice patterns for ophthalmic diseases [4,5,8].

Health claims databases are extensively utilized in retinal disease research due to their comprehensive records of healthcare practices and outcomes [4,5,8]. These databases typically include large-scale collections of data, such as the Medicare claims database, which provides insights into treatments and outcomes for a significant portion of the elderly population, and private insurer databases, which cover a diverse demographic [9]. Each database has unique advantages and limitations; therefore, the researcher’s choice of database for analysis is significant for achieving a specific research goal [10]. Researchers select these databases based on specific criteria, such as the age group, the geographical coverage, and the depth of data on specific healthcare services, which are crucial for studies on prevalence and treatment efficacy of retinal diseases.

### 2.1. Methodologies of Health Claims Database-Based Research

#### 2.1.1. Definition and Types of Health Claims Databases

Health claims databases are structured repositories that systematically collect and store healthcare transaction data primarily for billing and reimbursement purposes [1,4,9]. These databases are integral to the healthcare system, capturing detailed information about patient diagnoses, treatments, healthcare provider interactions, and the associated costs [1,4,9]. The data are typically sourced from healthcare providers, who submit claims to insurance companies or government payers for services rendered. There are two primary types of health claims databases: open and closed claims databases [11]. Open claims databases contain transactions that are still under review or pending resolution, providing real-time insights into ongoing healthcare activities. Closed claims databases, on the other hand, include transactions that have been finalized and fully processed, offering a complete view of healthcare events after all adjustments and reimbursements have been made [11].

Sources of health claim data are diverse, ranging from private insurance companies and pharmacy benefit managers to public entities like the Centers for Medicare and Medicaid Services (CMS). In some regions, such as the United States, all-payer claims databases (APCDs) aggregate data from multiple payers, providing a comprehensive view of healthcare utilization across different insurance types and patient demographics [12]. In Asia, health claims databases often integrate with other types of medical data, such as electronic medical records and laboratory data, to enhance the depth of information available for research and policy making [13]. For instance, in Japan, the DPC (Diagnosis Procedure Combination) system collects standardized medical treatment information, which is used alongside traditional health claim data to improve healthcare delivery and policy evaluation [14]. Similarly, in Europe, health claims databases are utilized not only for billing but also for monitoring public health trends and evaluating healthcare interventions across various countries, adapting to the diverse healthcare systems and regulatory environments present in the region [15].

Table 1 lists various health claims databases across different countries. 

Examples of health claims databases include:

Medicare Databases: Managed by the Centers for Medicare and Medicaid Services (CMS), these databases contain claims data for beneficiaries enrolled in Medicare, the federal health insurance program for individuals aged 65 and older or with certain disabilities. Medicare databases include information on hospital stays (Part A), outpatient services (Part B), and prescription drugs (Part D).

Medicaid Databases: Also managed by CMS, Medicaid databases capture claims data for Medicaid, the joint federal–state health insurance program for low-income individuals and families. These databases provide comprehensive data on a wide range of healthcare services utilized by Medicaid beneficiaries.

Commercial Claims Databases: Compiled by private health insurance companies, these databases contain claims data for individuals covered under employer-sponsored or individually purchased health plans. Examples include:

FAIR Health National Private Insurance Claims (NPIC) database: Includes information on medical and dental claims from private insurers nationwide.

German Pharmaco-epidemiological Research Database (GePaRD): Provides detailed claims data in Germany.

VEKTIS: Collects comprehensive health claims data in the Netherlands.

JMDC Claims Database, Medical Data Vision (MDV) Database, Nihon-Chouzai Pharmacy Claim Database: Provides extensive health claims data in Japan.

APCDs: These state-level databases include medical, pharmacy, and dental claims, as well as eligibility and provider files from both private and public payers. APCDs are designed to provide comprehensive data on healthcare utilization, costs, and quality across different insurance types within a state. Examples include Securite Sociale de l’Assurance Maladie (SNIIRAM) and Echantillon Généraliste de Bénéficiaires (EGB) in France, Agencia Regionale di Sanita Tuscany database (ARS) in Italy, National Database of Health Insurance Claims (NDB) in Japan, National Health Insurance Research Database (NHIRD) in Taiwan, Integrated Health Information Systems (IHiS) and National Electronic Health Record (NEHR) in Singapore, National Health Fund in Poland, Health Insurance Review and Assessment Service (HIRA) Database in South Korea, and Pharmaceutical Benefits Scheme (PBS) in Australia.

#### 2.1.2. Data Elements and Variables in Claims Data

Health claims databases typically include a variety of data elements and variables that are essential for constructing healthcare measures and conducting research. These elements can be broadly categorized into the following groups: patient demographics, medical claims, pharmacy claims, enrollment information, provider details, and cost information (Table 2) [5,57]. Health claims databases capture a wide range of data, which are used to understand population characteristics, healthcare encounters, medication use, insurance coverage, practice patterns, and the financial aspects of healthcare services. These datasets enable researchers to conduct comprehensive analyses, such as stratifying populations by demographic factors, identifying treatment patterns, assessing medication adherence, analyzing healthcare delivery, and evaluating economic impacts of healthcare interventions (Table 2).

The collection methods for health claim data involve the aggregation of electronic records from various healthcare and insurance platforms. These records are generated when healthcare providers submit claims to payers, such as insurance companies or government programs, like Medicare and Medicaid. The data typically include standardized diagnostic codes (ICD-10), procedure codes (CPT), and national provider identifiers (NPI), which ensure uniformity and facilitate large-scale data analysis. Claims data are recorded at the time of patient–provider interactions, ensuring that the information reflects real-world clinical practice. However, the accuracy and completeness of claims data can be influenced by factors such as coding practices, billing errors, and variations in data submission processes [58].

### 2.2. Strengths and Trends of Health Claims Database-Based Studies 

Health claims databases offer several key strengths that have driven their widespread adoption for real-world research in various therapeutic areas [59], including retinal diseases. These strengths, along with recent trends, are presented in Table 3. 

#### 2.2.1. Strengths 

One of the primary advantages of health claims databases is their ability to provide large sample sizes, often comprising millions of patients across diverse demographic and geographic regions [5]. This large-scale data enables robust statistical analyses, particularly for studying rare conditions or subgroups that may be underpowered in traditional clinical trials.

Claims databases capture longitudinal data on patient interactions with the healthcare system over extended periods, allowing researchers to track disease progression, treatment patterns, and long-term outcomes [4]. This longitudinal view is invaluable for understanding the real-world course of chronic conditions such as retinal diseases.

Unlike tightly controlled clinical trials, claims data reflect actual clinical practice, providing insights into real-world practice patterns, adherence, and effectiveness across diverse patient populations and care settings [41,60,61]. This real-world evidence complements data from clinical trials and can inform clinical decision-making [61].

Additionally, claims data contain detailed information on healthcare resource utilization, costs, and reimbursement, enabling analyses of economic burden, cost-effectiveness, and healthcare delivery patterns [62]. Such analyses are crucial for informing healthcare policy and resource allocation decisions.

#### 2.2.2. Recent Trends and Growth

The use of health claims databases for research has seen significant growth in recent years, driven by the increasing availability and accessibility of these data sources, as well as the recognition of their value in generating real-world evidence. Technological advancements in data analytics and computing power have further facilitated the analysis of large-scale claims data [63].

Moreover, there is a growing trend towards integrating claims data with other real-world data sources, such as electronic health records (EHRs) and patient-reported outcomes, to provide a more comprehensive view of clinical outcomes. Additionally, the formation of multi-database research networks and consortia has enabled collaborative research efforts and the pooling of data from multiple sources, further enhancing the statistical power and generalizability of claims database studies [64].

Therefore, health claims databases offer substantial strengths in terms of large sample sizes, longitudinal data capture, insights into real-world practice, and the ability to study healthcare utilization and costs. These strengths, coupled with recent trends towards data integration and collaborative research efforts, have solidified the role of claims databases as a valuable source of real-world evidence in retinal diseases and other therapeutic areas. 

### 2.3. Study Designs in Health Claims Database-Based Studies

#### 2.3.1. Observational Studies

Observational studies using health claims databases are a cornerstone in real-world research for retinal diseases [65]. Prospective vs. retrospective and cross-sectional vs. longitudinal are the key features of observational studies. These studies involve monitoring patients from the researchers, allowing for the natural progression of the retinal diseases of interest [66]. Observational studies are essential for understanding the epidemiology, treatment patterns, and outcomes in a real-world setting. They are particularly valuable for identifying associations and generating hypotheses regarding risk factors, disease progression, and the impact of different interventions on health outcomes [67]. Therefore, applications in retinal diseases includes natural history studies observing the progression of diverse retinal diseases over time, risk factor identification determining factors associated with the onset and progression of the diseases, and treatment pattern research examining the real-world use of various treatments and their outcomes. 

#### 2.3.2. Cohort Studies and Case-Control Studies

Cohort studies follow a group of individuals (cohort) over time to assess the incidence of outcomes and their association with exposures [68,69]. Cohort studies can be prospective (following individuals forward in time) or retrospective (using existing data to look back in time). Prospective cohort studies involve identifying a cohort and following them into the future to observe outcomes. They provide high-quality data on temporal relationships but can be time-consuming and expensive. However, retrospective cohort studies use existing data to identify a cohort and follow them backward in time. They are less costly and quicker to conduct than prospective studies but may suffer from incomplete or inaccurate historical data.

Cohort studies are particularly useful for studying the natural history of diseases and the long-term effects of exposures [68,69]. In the field of retinal disease research, this study design may be useful for studying the incidences of retinal diseases, progression rates, and long-term outcomes of treatments [70]. For example, a cohort of diabetic patients can be followed up to study the incidence of retinopathy and comparing those treated with different medications/treatments [70,71].

Case-control studies compare individuals with a specific outcome (cases) to those without the outcome (controls) to identify factors associated with the outcome [72,73]. They are particularly useful for studying rare diseases or outcomes and can be conducted relatively quickly and inexpensively. Therefore, applications may be ideal for studying outcomes or diseases with long latency periods, for example, identifying lifestyle or clinical risk factors by comparing diabetic patients with DR or diabetic macular edema (DME) to those without [74,75].

In case-control studies, accurate identification of cases and appropriate selection of controls are critical [76]. Controls should be representative of the population from which the cases arose and should not have the outcome of interest. Cases and controls can be matched on certain variables (e.g., age, sex, and systemic diseases) to control for confounding. Statistical adjustments (e.g., logistic regression) can also be used to account for differences between groups.

## 3. Applications of Real-World Research Using Health Claims Databases in Retinal Diseases

Real-world research using health claims databases provides critical insights into the epidemiology, effectiveness, and safety of treatments for diverse retinal diseases. These studies were applied for epidemiologic studies or effectiveness/safety research, in order to inform clinical practice, guide public health or treatment strategies, and ultimately improve patient outcomes for those with retinal diseases [77].

### 3.1. Epidemiological Studies

#### 3.1.1. Prevalence and Incidence of Retinal Diseases and Their Complications

Epidemiological studies using health claims databases provide valuable insights into the prevalence and incidence of retinal diseases, particularly DR, or its complications. These studies leverage large, diverse populations to generate robust estimates that inform public health strategies and resource allocation. For example, the algorithm for identifying patients with DME using ICD-9-CM diagnosis codes demonstrated high sensitivity (0.88) and specificity (0.96), with excellent agreement with medical records (kappa = 0.84), suggesting that it can accurately identify DME in claims data [78].

Studies utilizing health claims databases have demonstrated that the nationwide incidence of retinopathy of prematurity (ROP) can be determined and trends can be assessed using national health claims data [79]. For instance, in South Korea, both the incidence of ROP and the percentage of infants with ROP undergoing treatment significantly decreased over a 12-year period [79]. A few Asian studies also investigated on the incidence of retinal vascular diseases [80,81]. Furthermore, a study of a population-based health claims database found that the presence of ROP and treatment for ROP were associated with significantly higher risks of complications, such as strabismus, amblyopia, and glaucoma, among premature infants [82].

Health claims databases have been used to estimate the prevalence and incidence of age-related macular degeneration (AMD) in several countries [83,84,85] These databases provided detailed estimates of both AMD and exudative AMD across multiple nationwide populations, which are useful for healthcare policymaking.

#### 3.1.2. Risk Factors and Predictors

Identifying risk factors and predictors for retinal diseases is essential for developing preventive strategies and personalized treatment plans [86,87]. Health claims databases provide a wealth of data that can be used to identify these factors [88]. For example, diabetes patients who developed advanced complications and required laser photocoagulation had significantly lower rates of healthcare utilization, including visits to general practitioners, HbA1c testing, HDL-cholesterol testing, and specialist consultations, compared to those who did not [89]. Another study showed that incidence of ROP largely depends on the gestational age at birth [79]. In AMD, the risk factors for advanced diseases included age, a history of smoking, and systolic blood pressure [90].

### 3.2. Effectiveness/Safety Research

#### 3.2.1. Evaluation of Efficacy for Treatments and Interventions

Comparative effectiveness research using health claims databases evaluates the real-world efficacy of various treatments and interventions [62,64], which can be potentially utilized for retinal diseases. This research is critical for informing clinical practice and optimizing patient outcomes. 

The introduction of anti-vascular endothelial growth factor (VEGF) agents, such as ranibizumab and aflibercept, has revolutionized the treatment of several retinal diseases, including AMD, DME, and DR. Studies using Medicare data have shown a significant increase in the use of these therapies following FDA approval, with corresponding improvements in patient outcomes [91]. Another study that analyzed Medicare Part B data from 2012 to 2015 found an increasing trend in the use of anti-VEGF therapies, particularly aflibercept. In that study, the number of bevacizumab and ranibizumab injections decreased, coinciding with a 69.4% increase in aflibercept injections [92].

In addition to anti-VEGF agents, intravitreal steroid injections have emerged as an important treatment option for various retinal diseases, particularly DME. However, specific health claims database studies on intravitreal steroids are limited. Therefore, comparative studies should be performed to compare the efficacy of different anti-VEGF agents and between anti-VEGF agents and intravitreal steroids, to provide insights into the optimal therapy selection in clinical practice for diverse retinal diseases.

#### 3.2.2. Safety Research

Safety research is essential for understanding the risks associated with different treatments and ensuring patient safety. Health claims databases offer a comprehensive view of adverse events and complications in real-world settings [93,94].

For example, Sultana et al. compared the risk of non-ocular hemorrhage between intravitreal aflibercept and ranibizumab as well as between these anti-VEGF drugs and intravitreal dexamethasone. A retrospective cohort analysis of Italian claims databases from 2011 to 2016 found no increased risk of non-ocular hemorrhage associated with aflibercept compared with ranibizumab over 180 days of follow-up, and a similar risk when compared with dexamethasone [95].

Studies have assessed the ocular safety profiles of various systemic medications such as pentosan polysulfate and hydroxychloroquine and intraocular treatment [40,41,60,96,97]. For example, research has examined the incidence of complications such as endophthalmitis and retinal detachment following cataract surgery or intravitreal anti-VEGF injections [98,99,100]. Comparative safety research may also evaluate the relative risks of different treatments. 

### 3.3. Health Economics and Burden of Disease

Real-world research using health claims databases provides comprehensive insights into the healthcare utilization, costs, and economic impact of diseases [101,102,103]. Healthcare utilization and costs associated with retinal diseases can be significant and vary by disease severity. Studies have shown that individuals with diabetic complications, such as DR, incur substantial healthcare costs due to frequent medical visits, treatments, and management of complications [104,105,106]. The economic burden of retinal diseases includes direct medical costs and indirect costs (e.g., productivity losses associated with vision impairment). Direct costs include expenses related to medical treatments, hospitalizations, and outpatient visits. For example, the cost of anti-VEGF therapy for several retinal diseases, such as age-related macular degeneration and DR can be substantial, with studies reporting a wide range of treatment costs depending on the specific medication and treatment regimen [107]. The financial impact is further exacerbated by the need for ongoing management and the potential for severe complications.

When considering the societal burden of retinal diseases, the impact of visual impairment must be carefully considered, as many of these conditions significantly affect vision. The CDC estimates that the total economic burden of vision loss and blindness in the U.S. is $134.2 billion annually, with medical, nursing home, and supportive services accounting for $98.7 billion of this total [108]. The economic impact of retinal diseases extends beyond direct healthcare costs to include broader societal costs, such as lost productivity and reduced quality of life [109].

The real-world economic burden of retinal diseases varies by disease and country. For example, inherited retinal diseases impose a significant societal burden in many countries due to the long-term effects of vision loss, reduced productivity, and the costs of treatments, such as gene therapy and retinal prostheses [110]. However, this burden can change over time and should be investigated extensively in future studies using health claims databases, which can provide insights into costs, healthcare utilization, and trends over time in a large number of patients. 

### 3.4. Practice Patterns and Quality of Care

Practice pattern research examines how healthcare providers manage patients with retinal diseases and the extent to which clinical guidelines are followed. Studies have shown that appropriateness of eye care delivery widely varied in diabetic eye care and adherence to clinical guidelines varies among providers [111]. One study found higher rates of the first retinopathy intervention and vitrectomy in patients continuing antidiabetic medication than in those newly initiating it [112]. Older age, insulin use, and dependent status were associated with a higher incidence of these interventions [112]. Another study found that a significant proportion of newly diagnosed type 2 diabetes patients in Germany did not follow recommended DR screening guidelines, with half not visiting an ophthalmologist within 2.25 years of diagnosis [113]. Older age, higher disability levels, and male sex were associated with lower screening uptake, while enrollment in a diabetes management program significantly increased the likelihood of screening. Scondotto et al. examined the use of intravitreal anti-VEGF and dexamethasone in four Italian regions from 2010 to 2016, highlighting the regional differences in drug prevalence and treatment practices [114]. Despite increasing drug use over time, the findings suggest variability in adherence to the recommended dosing intervals, indicating the need for improved clinical practices to optimize treatment benefits [114]. This field of research is particularly advantageous for using health claims databases as they provide large, real-world data sets that can reveal patterns and outcomes in patient care, enabling a better understanding of adherence to clinical guidelines and identifying areas for improvement.

Furthermore, quality indicators and healthcare disparities are critical components of evaluating and improving the care provided to patients with retinal diseases. Key quality indicators for DR care include the frequency of eye examinations, timely treatment of vision-threatening conditions, and patient adherence to follow-up appointments [41,60]. Monitoring these indicators helps assess the quality of care and identify gaps in service delivery. Disparities in the prevalence, screening, and treatment of DR can be revealed, particularly among underserved and minority populations, using health claims databases. Social determinants of health, such as socio-economic status and access to healthcare, may play a significant role in these disparities, which can be also assessed using the database.

## 4. Challenges and Considerations

The challenges and limitations of using health claims databases in medical research are outlined in Table 3. 

### 4.1. Data Limitations and Biases

#### 4.1.1. Inaccuracy of Data and Lack of Specific Codes 

Health claims databases, while invaluable for real-world medical research and economic analysis, have notable limitations, particularly regarding data accuracy and the lack of specific codes for certain conditions. Inaccuracies can arise from various sources, such as errors in data entry, misclassification of diseases, and inconsistencies in coding practices among different healthcare providers and institutions. These inaccuracies can lead to flawed research outcomes and misinform healthcare policy and practice. Additionally, the lack of specific diagnostic and procedural codes for some conditions can pose significant challenges. Health claims databases often rely on standardized coding systems, like the International Classification of Diseases (ICD), which may not always have precise codes for emerging or less common medical conditions. This can result in the underreporting or misclassification of these conditions, further compromising the reliability of the data. As a result, studies based on these databases may not fully capture the true prevalence/incidence and impact of certain diseases, necessitating the need for additional data sources or more refined coding systems to improve data quality and accuracy.

#### 4.1.2. Selection Bias and Confounding Factors

Selection bias and uncontrolled confounding factors are significant challenges in using health claims databases for research [115]. Selection bias occurs when the study population is not representative of the general population, leading to skewed results. For example, health claims databases often exclude uninsured individuals, which can potentially bias the results obtained from the study.

Confounding factors are variables that are related to both the exposure and the outcome, potentially distorting the true relationship between them. In claims data, confounding can arise from differences in patient demographics, comorbidities, and healthcare access. Researchers must use statistical methods, such as multivariable regression, propensity score matching, and stratification to control for these confounders.

#### 4.1.3. Generalizability and External Validity

Generalizability refers to the extent to which study findings can be applied to broader populations. Health claims databases often contain data from specific populations, such as those covered by Medicare, Medicaid, or private insurance, which may not be representative of the entire population. This limitation affects the external validity of the research, making it difficult to generalize findings to other groups, such as the uninsured or those in different geographic regions.

To enhance generalizability, researchers should consider the demographic and clinical characteristics of the study population and compare them with the target population. Additionally, using multiple databases and combining claims data with other data sources might improve the representativeness of the findings.

### 4.2. Privacy and Ethical Concerns

Data privacy regulations, such as the Health Insurance Portability and Accountability Act (HIPAA) in the United States and the General Data Protection Regulation (GDPR) in the European Union, impose strict requirements on the use and disclosure of health information. These regulations aim to protect patient privacy and ensure that personal health information is used responsibly.

Compliance with these regulations involves implementing measures to de-identify data, obtain necessary consents, and ensure secure data storage and transmission [116]. Researchers must navigate these regulatory frameworks to access and use health claims data while maintaining patient confidentiality and privacy.

Informed consent is a fundamental ethical requirement in research, ensuring that participants are aware of and agree to the use of their data. However, obtaining informed consent for the use of health claims data might be almost impossible, especially when dealing with large, retrospective datasets. To address this, researchers usually rely on de-identified data, where personal identifiers are removed to protect patient anonymity, which mitigates privacy concerns.

### 4.3. Methodological Issues and Validation

Health claims databases present significant challenges related to methodological issues. Addressing these challenges through rigorous validation, advanced analytical techniques, and transparent reporting is essential for producing reliable and impactful research findings. Validation studies are essential for assessing the accuracy and reliability of health claim data. These studies compare claims data with other data sources, such as medical records, to verify the correctness of diagnoses, procedures, and other key variables. Validation helps identify potential biases and errors in the data, enhancing the credibility of research findings. Sensitivity analyses involve testing the robustness of study results by varying key assumptions and parameters [117]. This approach helps identify the impact of potential biases and uncertainties on the findings, providing a more comprehensive understanding of the data’s reliability.

Addressing the limitations and uncertainties inherent in health claims data is crucial for valid and reliable research on retinal diseases. Researchers should acknowledge the limitations of their data, such as incomplete or inaccurate coding, and discuss how these limitations may affect their findings.

Combining claims data with other data sources, such as EHRs, registries, and patient-reported outcomes, can provide a more comprehensive view of patient care and outcomes. Providing clear and detailed descriptions of data sources, study design, and identification and analytical methods in retinal disease research helps ensure transparency and reproducibility. Researchers should also report any potential biases and limitations and discuss their implications for the study’s conclusions.

## 5. Future Directions

The future of real-world research using health claims databases for retinal diseases is promising, with advancements in data collection and analysis, integration with EHR and other health databases, collaborative research networks, and supportive regulatory policies. These developments will enhance our understanding of healthcare delivery and outcomes, ultimately leading to improved patient care and health system performance.

### 5.1. Advancements in Data Collection and Analysis

Advancements in data collection and analysis are poised to significantly enhance the utility of health claims databases for retinal disease research. The application of big data analytics allows for the processing and analysis of vast amounts of health claim data, uncovering patterns and insights that were previously unattainable. Techniques such as machine learning and artificial intelligence (AI) can be used to predict patient outcomes, identify risk factors, and optimize treatment strategies. 

Integrating EHR data with claims data is an important step for advancing data analysis. More specifically, the integration of health claims databases with EHRs offers a more comprehensive view of patient care and outcomes. Linking claims and EHR data at the patient level using unique identifiers allows for the creation of rich, longitudinal datasets that capture both clinical and administrative information. This approach provides a holistic view of patient’s healthcare and facilitates more robust real-world studies. Another recent article reviewed the methodological challenges in assessing the systemic safety of intravitreal anti-VEGF drugs using RWD, such as spontaneous reporting systems, EHR, and claims databases [118]. This highlights the need for careful selection of data sources and discusses the complementary strengths and limitations of various analytical methods, advocating their combined use to provide a more balanced risk assessment.

Furthermore, implementing bidirectional data exchange mechanisms between EHRs and claims databases can enhance data accuracy and completeness. This allows for real-time updates and more dynamic research capabilities. 

### 5.2. Collaborative Research Networks and Consortia

Collaborative research networks and consortia play a crucial role in advancing real-world research by pooling resources and expertise. Networks such as the Patient-Centered Outcomes Research Network (PCORnet) and the FDA Sentinel Initiative facilitate large-scale, multi-institutional studies by integrating data from various sources, including claims and EHRs [119]. These networks enhance the statistical power and generalizability of research findings. Consortia bring together stakeholders from academia, industry, and government to address common research challenges and develop standardized methodologies. Collaborations between public health agencies and private sector organizations can enhance data integration and analysis, leading to more effective and efficient healthcare solutions for patients with retinal diseases.

### 5.3. Regulatory Landscape and Policy Implications

The regulatory landscape and policy environment significantly influence the use of health claims databases for research. Key considerations include data privacy regulations, policy incentives, standardization efforts, and ethical considerations [3,120].

Compliance with data privacy regulations, such as HIPAA and GDPR, is essential for protecting patient information and maintaining public trust. Researchers must navigate these regulations to ensure ethical and legal use of health claims data for retinal disease research. Regulatory bodies can promote the development and adoption of data standards to facilitate interoperability and data sharing across different healthcare systems. This can improve the quality and consistency of data used in real-world research. Ensuring patient anonymity is also critical ethical considerations in the use of health claim data for retinal disease research.

## 6. Conclusions

This review has highlighted the significant role of health claims databases in advancing real-world research on retinal diseases. Health claims databases provide robust data on the prevalence and incidence of common retinal diseases, particularly DR and AMD, across diverse populations. These databases enable the identification of risk factors and predictors, contributing to a better understanding of disease epidemiology. Real-world evidence from health claims databases has been instrumental in evaluating the effectiveness and safety of various treatments for retinopathy. Additionally, the economic impact of retinopathy, including healthcare utilization and costs, is substantial. Health claims databases facilitate comprehensive cost analyses, highlighting the financial burden of the disease and informing healthcare policy and resource allocation. Moreover, real-world research using health claims databases provides valuable insights into patient outcomes, practice patterns, and quality indicators. These studies help identify gaps in care and disparities, guiding efforts to improve healthcare delivery and equity. The role of health claims databases in retinopathy research is expected to grow, driven by advancements in data collection, integration, and analysis. The integration of claims data with EHR and other real-world data sources will provide a more comprehensive view of patient care and outcomes. 

In conclusion, health claims databases play an important role in conducting real-world studies on retinal diseases. By addressing current challenges and leveraging future opportunities, researchers can generate impactful evidence that enhances clinical practice, informs policy, and ultimately improves the quality of life in patients with the diseases.

## Figures and Tables

**Table 1:** Examples of health claims databases in different countries.

Country	Health Claims Databases	References
USA	Medicare	[16,17,18]
	Medicaid	[19]
	CMS Chronic Conditions Data Warehouse (CCW)	[20]
	Truven Health MarketScan^®^ Databases	[21]
	Optum’s Clinformatics^®^ Data Mart	[22]
	Komodo’s Healthcare Map^TM^	[23]
Japan	National Database of Health Insurance Claims (NDB)	[24,25]
	JMDC Claims Database	[26,27]
	Diagnosis Procedure Combination (DPC) Database	[28,29]
	Medical Data Vision (MDV) Database	[30,31]
	Nihon-Chouzai Pharmacy Claim Database (less known)	
Taiwan	National Health Insurance Research Database (NHIRD)	[32,33]
Australia	Medicare Benefits Schedule (MBS)	[34,35]
	Pharmaceutical Benefits Scheme (PBS)	[36]
	Centre for Health Record Linkage (CHeReL)	[37,38]
	HealthLinQ (less known)	
	Victorian Data Linkages (VDL) (less known)	
	SA-NT DataLink	[39]
South Korea	Health Insurance Review and Assessment Service (HIRA) Database	[40,41]
	National Health Insurance Service (NHIS) Database	[42]
Thailand	EHMIS (less known)	
Malaysia	United Nations University-Casemix (UNU-Casemix) Database (less known)	
France	Securite Sociale de l’Assurance Maladie (SNIIRAM)	[43]
	Echantillon Généraliste de Bénéficiaires (EGB)	[44,45]
Germany	German Pharmaco-epidemiological Research Database (GePaRD)	[46,47]
Italy	Agencia Regionale di Sanita Tuscany database (ARS)	[48,49]
	Hospital Information System—Lazio (HIS)	[50]
	Region Emilia Romagna Database (RER)	[51,52]
	Caserta Database	[53]
Netherlands	VEKTIS database	[54]
Poland	National Health Fund database	[55]
Hungary	National Health Insurance database	[56]

**Table 2:** The key categories of data captured in health claims databases, the specific types of data within each category, and their primary uses in research and analysis.

Category	Data	Uses
Patient Demographics	Age, gender, geographic location, enrollment periods	Understanding population characteristics, stratifying analyses by demographic factors
Medical Claims	Diagnosis codes (ICD-9/ICD-10), procedure codes (CPT/HCPCS), service dates, provider information	Identifying healthcare encounters, treatments, and procedures
Pharmacy Claims	National Drug Codes (NDCs), prescription dates, days supplied, quantities dispensed	Providing information on medication use and adherence patterns
Enrollment Information	Plan type, coverage periods, payer information	Understanding insurance coverage and eligibility status of patients
Provider Details	Specialty, facility type, geographic location	Analyzing practice patterns and healthcare delivery
Cost Information	Paid amounts, deductibles, copays, coinsurance	Conducting economic analyses and understanding the financial burden of healthcare services

**Table 3:** Summary of the strengths and limitations of health claims database-based studies.

Strengths	Limitations
Large sample sizes across diverse patient populations	Lack of detailed clinical information
Longitudinal data capture over extended periods	Inaccuracy of data
Insights into real-world treatment patterns and outcomes	Limited information into appropriateness of healthcare or its utilization
Ability to study healthcare utilization and costs	Data fragmentation across providers and payers
Recent growth and increasing availability	Absence of certain data types (labs, imaging, genetic data)
Integration with other real-world data sources (EHR)	Lack of specific codes for some conditions
Formation of multi-database research networks/consortia	Potential selection bias due to exclusion of uninsured patients and cash transactions
